# Serological Survey of *Ehrlichia canis* and *Anaplasma phagocytophilum* in Dogs from Central Italy: An Update (2013–2017)

**DOI:** 10.3390/pathogens8010003

**Published:** 2019-01-04

**Authors:** Valentina Virginia Ebani

**Affiliations:** Department of Veterinary Science, University of Pisa, viale delle Piagge 2, 56124 Pisa, Italy; valentina.virginia.ebani@unipi.it

**Keywords:** *Ehrlichia canis*, *Anaplasma phagocytophilum*, dogs, seroprevalence

## Abstract

*Ehrlichia canis* and *Anaplasma phagocytophilum* are tick-borne bacteria of veterinary concern. Indirect immunofluorescent assay was carried out to detect antibodies against *E. canis* and *A. phagocytophilum* in 1026 owned dogs living in Central Italy during the period 2013–2017. One hundred and eighty-six (18.12%) dogs were positive for at least one pathogen and 14 (1.36%) for both agents. More in detail, 166 (16.18%) samples were positive for *E. canis* and 34 (3.31%) for *A. phagocytophilum*. No statistically significant differences in the seroprevalence values related to gender were detected, whereas the highest rate to *E. canis* occurred in animals aged more than 10 years. Mean seroprevalence values for both *E. canis* and *A. phagocytophilum* detected in 2014 and 2015 were statistically higher with respect to other years. Even though dogs’ owners are informed about the risk of pet infections by tick-borne pathogens and prophylaxis against ticks is often executed, *E. canis* and *A. phagocytophilum* are still present and infect the canine population in Central Italy.

## 1. Introduction

*Ehrlichia canis* and *Anaplasma phagocytophilum* are obligate intracellular Gram-negative bacteria belonging to the family Anaplasmataceae, order Rickettsiales [1]. Both pathogens are responsible for vector-borne infectious diseases of veterinary concern. *E. canis* mainly parasitizes the cells of the mononuclear phagocyte system and causes the canine monocytic ehrlichiosis (CME) characterized by clinical and hematological abnormalities such as fever, anorexia, vomiting, diarrhea, lymphadenopathy, petechial hemorrhages, bleeding tendency, anemia, and thrombocytopenia. CME after a subclinical or acute form can determine chronic infection that can persist for years [2].

*E. canis* usually infects dogs, but it is also able to cause infection in other canids and cats [3,4]. Moreover, a role as zoonotic agent has been supposed after the detection of this pathogen in clinical samples of human beings with clinical signs similar to those of CME [5].

*A. phagocytophilum* has tropism for granulocytes, mainly neutrophils and it is able to infect dogs, other canids, cats, horses, domestic and wild ruminants [6]. *A. phagocytophilum*-infected dogs may have clinical forms varying in severity degree. In general, *A. phagocytophilum* seems to cause less severe clinical signs than *E. canis*. Dogs may be asymptomatic or show fever, lethargy, reluctance to move, vomiting, diarrhoea, and nervous system dysfunction [7].

*A. phagocytophilum* is a zoonotic agent, responsible for the human granulocytic ehrlichiosis (HGE) or anaplasmosis (HGA) characterized by influenza-like symptoms that on rare occasions could have a fatal conclusion [8].

Both *E. canis* and *A. phagocytophilum* are transmitted by ticks during their blood meal.

*Rhipicephalus sanguineus* sensu lato (s.l.), known as the brown dog-tick, is considered the main vector of *E. canis* as well as the only vector involved in the transmission of *E. canis* in Europe [2], even though *Dermacentor variabilis*, the American dog tick, has been shown to be a capable vector [9].

*Ixodes ricinus* ticks are considered the main vectors of *A. phagocytophilum* in Europe. Moreover, vector competence has been proven for other *Ixodes* species in other geographic areas. *A. phagocytophilum* DNA has been detected in *Dermacentor* spp., *Haemaphysalis* spp. and *Amblyomma americanum* that are supposed, but not proven, to be involved in the transmission of this microorganism [6].

*A. phagocytophilum* and *E. canis* have a worldwide distribution and previous investigations demonstrated their presence among canine population in Italy, too [10,11,12,13,14,15,16,17,18].

Data about the prevalence of these vector-borne pathogens are very variable in relation to the methods employed for their detection, geographic area, climatic conditions, presence of hematophagous vectors, environment in which dogs live, prophylaxis against arthropods. Moreover, available data are strictly related to animals’ characteristics such as attitude (for instance pets or hunting dogs), free-roaming or owned, symptomatic or asymptomatic.

The aim of the present study was to update the information about the seroprevalence of *E. canis* and *A. phagocytophilum* infections among dogs living in Central Italy during a five-year period from 2013 to 2017.

## 2. Results

Among the 1026 examined dogs, 186 (18.12%) were serologically positive for at least one pathogen and 14 (1.36%) for both agents. More in detail, 166 (16.18%) samples were positive for *E. canis* and 34 (3.31%) for *A. phagocytophilum*. The number of dogs positive for both pathogens at the different antibody titers is reported in Table 1.

No statistically significant differences in the seroprevalence values related to gender were detected. Considering the animals’ age, the highest percentage of *E. canis*-positive dogs was observed in dogs aged more than 10 years, whereas there were no significant differences in the prevalence for *A. phagocytophilum* (Table 2).

Mean seroprevalence values detected in 2014 and 2015 were statistically higher (χ^2^ test, *p* < 0.05) than those observed in the other years, as reported in Table 3.

## 3. Discussion

The mean seroprevalences detected for *E. canis* and *A. phagcoytophilum* show the presence of both tick-borne pathogens among the canine population living in Central Italy in the period from 2013 to 2017, even though the seropositive reactions could be due not only to current infections, but also to previous exposure to the studied microorganisms.

*E. canis* was the most widespread tick-borne pathogen, with a mean seroprevalence of 16.18%. No significant differences were detected in relation to gender of the tested dogs, whereas seroprevalence values were different in relation to the age; in particular, the highest percentage of seropositive reactions was found in the oldest animals. This result is in agreement with those of other surveys [19] and could be related to increasing vector exposure depending on animals’ age and/or immunological status [20,21].

Seroprevalence for *A. phagocytophilum* was significantly lower with a mean value of 3.31%, and no statistically significant differences were observed in relation to age and gender of the analyzed dogs.

As has been suggested by other authors [19], the positive reactions to *A. phagocytophilum* could be due to cross-reactivity with *Anaplasma platys*, which has been previously found in the canine population in Italy [22,23].

Moreover, cross-reactions between antibodies against different *Ehrlichia* species can occur. In fact, antibodies against *E. canis* may cross-react with antigens of *Ehrlichia ewingii*, *Ehrlichia chaffeensis*, *Ehrlichia ruminantium*, *Neorickettsia sennetsu* (formerly *Ehrlichia sennetu*), and *Neorickettsia risticii* (formerly *Ehrlichia risticii*) [24,25]. However, the presence of these pathogens in Italy has not been documented, suggesting that they do not interfere in the serological diagnosis.

A low percentage of dogs (1.36%) were seroreactive to both *E. canis* and *A. phagocytophilum* antigens. Some authors suggest that cross-reactions can occur between these agents, particularly when one of the two pathogens causes very high titers or when the follow up is prolonged [24]. In this study, dogs resulted positive to both pathogens with similar antibody titers, thus it cannot be excluded that animals had been exposed to both vector-borne microorganisms.

Limited data on seroprevalence of these tick-borne infections in Italian canine population are available. Moreover, it is difficult to compare seroprevalences detected in the present survey to values observed in other studies, because canine population and geographic area are very different.

However, some previous investigations found values in agreement with our results. A recent study performed among hunting dogs from Southern Italy found seroprevalences for *E. canis* and *A. phagocytophilum* of 7.6% and 4.4%, respectively [18]. Another survey on candidate blood donors and free-roaming dogs from Northeastern Italy detected seroprevalences for *A. phagocytophilum* of 4.7% and 3.3%, respectively. For *E. canis* this value was 0.9% in free-roaming dogs, while all candidate donors were negative [26].

A similar investigation carried out on dogs living in Central Italy during the period 2008–2012 found lower mean seroprevalence for *E. canis* (7.07%), whereas seroprevalence for *A. phagocytophilum* was slightly higher (4.68%) [27].

The present study found statistically significant differences among the seroprevalence values for both investigated pathogens in relation to the year of sampling had been collected. In fact, the highest seroprevalences for *E. canis* were observed during 2014 (28.0%) and 2015 (20.4%), whereas the values significantly decreased in the following years.

A similar behavior was observed with the seroprevalences for *A. phagocytophilum*, with the highest values detected in 2014 (6.22%) and 2015 (5.1%).

These results could be related to the climatic conditions, that are known to strongly influence the presence of ticks and other hematophagous arthropods in the environment and consequently the vector-borne pathogens’ spreading.

The Italian Institute for Environmental Protection and Research (ISPRA) reports that 2014 and 2015 were very warm and rainy years in Italy. In more details, the average annual temperature in 2014 was 1.63 °C higher than the normal value in Central Italy. Moreover, 2014 total annual rainfall in Italy was overall higher than the climatic average of about 13% (12% in Central Italy) [28].

Climatic data for 2015 by the ISPRA show that the 2015 average temperature value in Italy was the highest since 1961, slightly higher than that of in 2014. In detail, the average annual temperature was 1.70 °C higher in Central Italy. The annual rainfall in Italy was overall lower than the climatic average of about 13%, but the climate in Central Italy was rainier than usual in March, August, and October [29].

The main vector of *E. canis* in Europe is *R. sanguineus* s.l., which is largely present in the Mediterranean basin. These ticks require a minimum environmental temperature of about 6 °C and a good degree of humidity. Rainy but not cold winters allow ticks to survive, although when environmental temperatures are too low, ticks are able to hibernate sheltered in the cracks of buildings and kennels [30].

*I. ricinus* ticks are widely distributed in most northern and central European areas and it is the main vector of *A. phagocytophilum* in Europe. This species is present in several parts of the Mediterranean region, including Italy. It can be mainly found in mixed and deciduous forests, open pastures, and other areas with high humidity [31]. Its wide distribution is also related to a broad host range, including many mammalian species and birds [32].

All animals tested in the present investigation were owned dogs with different attitude, but none of them was employed in hunting activity. Even though owners were not able to give information on tick infestation, it can be supposed that dogs had been in contact with *R. sanguineus*, *I. ricinus,* and probably other ticks in gardens, urban parks, and peri-urban recreational areas.

## 4. Material and Methods

### 4.1. Animals

From January 2013 to December 2017, peripheral whole-blood samples were collected from 1026 dogs. Animals were privately-owned dogs from different areas of Central Italy. No hunting dogs were included in the study. Moreover, animals were excluded if they were evaluated for suspected vector-borne diseases or if undergoing antibiotic treatment.

Once received, all samples were given an identification number and catalogued by data of sampling, animal age and gender. It was not possible to record data about tick infestation, because owners were not often able to give this information.

Whole-blood samples, drawn from the right or left cephalic vein, were centrifuged at 1500× g for 15 min. The sera were collected and immediately tested or stored at −20 °C.

#### Ethical statement

The collection of blood samples was executed for other diagnostic purposes by collaborating veterinarians during clinical visits. No dogs were submitted to the blood collection only for this study. However, in all cases informed consent was obtained from the owners.

### 4.2. Serological Analyses

The indirect immunofluorescence antibody test (IFAT) was executed on IFAT slides specific for *Anaplasma phagocytophilum* and *Ehrlichia canis* (Fuller Laboratories Fullerton, California, USA) antigens.

Blood sera were diluted 1:40 in phosphate-buffered saline (PBS, pH 7.2), considered as the cut-off dilution, and tested following the protocol previously reported [27]. Positive samples were two-fold serially diluted to determine the endpoint titre. Scores from 1 to 4 were assigned to the intensity of specific fluorescence and the antibody titre was defined as the major dilution with a 2 score.

### 4.3. Statistical Analysis

Statistical evaluation was carried out by the χ^2^ test to analyze the results of serological tests in relationship to gender and age of the examined dogs and to the years in which samples were collected. Values of *p* < 0.05 were considered significant.

## 5. Conclusions

Even though dog owners are informed about the risk of pet infections by tick-borne pathogens and prophylaxis against ticks is often executed, *E. canis* and *A. phagocytophilum* were still found to be present in Central Italy. For this reason, veterinarians should consider these two pathogens in the case of suggestive clinical signs and in routine health status checks. Climatic conditions, as suggested by the obtained results, may influence the spreading of ticks and consequently the presence of tick-borne pathogens in animal populations. Prevention against dog tick infestation through acaracide treatments is the main tool to prevent infections, and a prompt diagnosis is necessary for appropriate therapy.

Moreover, constant surveillance of animal populations (mainly pets which share the same environment with their owners) in order to verify the spread of arthropod-borne pathogens is necessary from an One Health perspective.

## Figures and Tables

**Table 1:** Number of dogs positive for *Ehrlichia canis* and *Anaplasma phagocytophilum* at the given antibody titer.

	Antibody Titers						
	1:40	1:80	1:160	1:320	1:640	1:1280	Total
*Ehrlichia canis*	68	32	38	24	1	3	166
*Anaplasma phagocytophilum*	10	3	18	2	1	-	34

**Table 2:** Serological results in relationship to gender and age of the canine study population.

Category		Study Population	*E. canis*-Positive Dogs (%)	*A. phagocytophilum*-Positive Dogs (%)
Gender	Male	588	95 (16.15)	18 (3.06)
	Female	438	71 (16.21)	16 (3.65)
Age	<1	41	4 (9.75)	1 (2.43)
	1–5	529	82 (15.50)	17 (3.21)
	6–10	411	69 (16.78)	15 (3.64)
	>10	45	11 (24.44)	1 (2.22)

**Table 3:** Seroprevalence of *Ehrlichia canis* and *Anaplasma phagocytophilum* among tested dogs in relationship to the different years.

Year	Number of Examined Dogs	*E. canis*-Positive Dogs (%)	*A. phagocytophilum*-Positive Dogs (%)
2013	197	26 (13.20)	5 (2.53)
2014	225	63 (28.0)	14 (6.22)
2015	196	40 (20.40)	10 (5.10)
2016	174	16 (9.19)	3 (1.72)
2017	234	21 (8.97)	2 (0.85)
TOTAL	1026	166 (16.18)	34 (3.31)
